# Effects of Three Commercially Available Sports Drinks on Substrate Metabolism and Subsequent Endurance Performance in a Postprandial State

**DOI:** 10.3390/nu9040377

**Published:** 2017-04-12

**Authors:** Lu Qin, Qi-Rong Wang, Zi-Long Fang, Ting Wang, Ai-Qi Yu, Yu-Jie Zhou, Yi Zheng, Mu-Qing Yi

**Affiliations:** 1 National Institute of Sports Medicine, National Testing & Research Center for Sports Nutrition, 1 Anding Road, Room 206, Chaoyang District, Beijing 100029, China; qinlu@link.cuhk.edu.hk (L.Q.); fangzilong@126.com (Z.-L.F.); fergie90@126.com (T.W.); 15201124762@163.com (A.-Q.Y.); jerrychou1209@sina.cn (Y.-J.Z.); zy065001@126.com (Y.Z.); muqingyi@163.com (M.-Q.Y.); 2 Department of Sports Science and Physical Education, The Chinese University of Hong Kong, Shatin, Hong Kong, China; 3 Department of Exercise Science, Arnold School of Public Health, University of South Carolina, Columbia, SC 29208, USA; 4 Sports Science College, Beijing Sport University, Beijing 100084, China; 5 School of Physical Education and Sports Science, South China Normal University, Guangzhou 510631, China

**Keywords:** non-fasted state, drinks, cycling, two session training, metabolism, time to exhaustion

## Abstract

**Purpose:** To examine the effects of commercially available sports beverages with various components on substrate metabolism and subsequent performance. **Methods:** Two studies were conducted in a double-blinded, counterbalanced manner. Study I was designed to determine the glycemic index, while study II determined the utilization of substrates and subsequent exercise performance. Ten healthy male participants (age 21.70 ± 2.41 years, height 176.60 ± 5.23 cm, weight 66.58 ± 5.38 kg, V̇O_2max_ 48.1 ± 8.4 mL/kg/min) participated in both study I and study II. Three types of commercially available sports beverage powders were used. The powders consisted primarily of oligosaccharides (low molecular weight carbohydrates, L-CHO), hydrolyzed starch (high molecular weight CHO, H-CHO), and whey protein powder with carbohydrate (CHO-PRO). They were dissolved in purified water with identical CHO concentration of 8% (*w*/*v*). In study I, each participant underwent two oral glucose tolerance tests (OGTT) and one glycemic response test for each sports drink. In study II, participants cycled for 60 min at 70% V̇O_2max_, one hour after consuming a standardized breakfast. One of four prescribed beverages (L-CHO, H-CHO, CHO-PRO, and Placebo control, PLA) was served at 0, 15, 30, 45 min during the exercise. Six hours after the first exercise session, participants came back for a “time to exhaustion test” (TTE). Blood samples were drawn at 0, 30, and 60 min in the first exercise session, while arterial blood gas analysis was conducted at 0, 30, and 60 min in both sessions. Subjective feelings (rating of perceived exertion and abdominal discomfort) were also evaluated every 30 min during exercise. **Results:** Compared to the reference standardized glucose solution, the glycemic index of the L-CHO beverage was 117.70 ± 14.25, while H-CHO was 105.50 ± 12.82, and CHO-PRO was 67.23 ± 5.88. During the exercise test, the insulin level at 30 and 60 min was significantly lower than baseline following the treatment of L-CHO, H-CHO, and PLA (*p* < 0.05). The CHO oxidation rate at 60 min in the first exercise session was significantly higher than that at 60 min in the second exercise session following the L-CHO treatment (*p* < 0.05). Time to exhaustion was not significantly different (*p* > 0.05). **Conclusion:** The CHO sports beverage with additional PRO maintains insulin production during endurance cycling at 70% V̇O_2max_ in the postprandial state. L-CHO sports beverage suppresses fat utilization during the subsequent exercise performance test. The subsequent exercise performance (as evaluated by TTE) was not influenced by the type of CHO or the addition of PRO in the commercially available sports beverages used in the present study.

## 1. Introduction

Termination of moderate to high intensity exercise—namely 65% to 85% V̇O_2max_—is greatly attributed to glycogen depletion and the reduced availability of carbohydrate (CHO) (e.g., blood glucose, CHO oxidation rate) [1]. CHO ingestion attenuated the decline in blood glucose and induced higher exogenous CHO oxidation rate at the late stage (>1 h) of exhaustive prolonged cycling at 71% VO_2peak_ [2]. Increases in both blood glucose and CHO oxidation rate following CHO ingestion are potentially beneficial for the sparing of muscle and liver glycogen [3]. Previous systematic review and meta-analysis also revealed that CHO supplementation enhances exercise performance, either evaluated by the method of time to exhaustion (TTE) or time trial (TT) [4]. TTE is measured at a predetermined exercise intensity, speed, or power output. TT is evaluated by either the time to complete a predetermined distance or amount of work or distance completed or work done in a predetermined time. Although there is a higher coefficient of variation in TTE than TT, TTE test is conducted in a certain fixed intensity. This allows less fluctuation in the exercise intensity and makes it feasible to compare the effect of different nutrition supplementations on physiological biomarkers during exercise [5]. 

Protein (PRO) is another type of commonly applied macronutrient that is normally co-ingested with CHO. Compared with an equal volume of CHO beverage (21.0 g CHO/355 mL), ingestion of CHO + PRO (53.0 g CHO + 14.0 g PRO/355 mL) enhanced glycogen repletion by 128% during the 4 h recovery following 2 h cycling exercise at 65%–75% V̇O_2max_. In addition, subsequent exercise performance—which was evaluated with TTE at 85% V̇O_2max_—was 55% greater than CHO beverage following the ingestion of CHO + PRO beverage [6]. In the study by Saunders et al. [7], they also compared the effect of the CHO + PRO beverage with CHO on exercise performance. Ingestion of CHO + PRO (7.3% CHO + 1.8% PRO) increased the TTE by 29% more than CHO (7.3% CHO) at 75% VO_2peak_ cycling exercise. 

Based on this evidence, most commercial sports beverages—including those involved in the chosen list of national sports teams—contains CHO and PRO. Among the different CHO types, low molecular weight CHO (L-CHO) has been considered to be more easily absorbed by the gastrointestinal system than high molecular weight CHO (H-CHO). The glycemic responses following ingestion of CHO was affected by different molecular size. In addition, the glycemic responses can also be affected when CHO is co-ingested with PRO. Glycemic response—frequently demonstrated as glycemic index (GI)—suggests the availability of CHO following the consumption of foods containing CHO. Kirwan et al. [8] compared the effect of nutrition supplementation with moderate glycemic index (MGI, GI = 61) with that of a high glycemic index (HGI, GI = 82), using the time to exhaustion cycling test at 60% V̇O_2max_. Although carbohydrate availability was more rapid following HGI than MGI, CHO and fat utilization were not different at the exhaustion point. There was also no significant difference between the two treatments. Another study [9] compared low GI (LGI, GI = 30) and HGI (GI = 72) foods for pre-exercise supplementation in a 40-km time-trial cycling test. The study found that LGI supplementation better preserved the CHO and lipid availability compared to HGI food. LGI supplementation reduced the time to complete the 40 km cycling distance by 3.2%. 

Athletes and others who want to improve their exercise performance need information on the effects of commercially available sports drinks on substrate metabolism and performance, especially with regard to real-world situations (e.g., training in a postprandial state). A systematic review revealed that carbohydrate ingestion may be beneficial for both time to exhaustion and time trial exercise performance longer than 70 min in the postprandial state [10]. However, little information exists comparing commercially available sports beverages with various characteristics (e.g., type of CHO, GI, with/without additional PRO). Moreover, there are normally more than one training sessions in one training day, indicating the necessity to investigate the role of those commercially available sports beverages on the subsequent exercise performance following the first training session. However, the effects of commercially available sports beverages with various CHO types and composition (e.g., CHO + PRO) on either energy substrate metabolism or subsequent exercise performance in a postprandial state have not been fully elucidated.

Therefore, this study was designed to examine the effects of three types of commercially available sports beverages on substrate metabolism and subsequent performance. The first session (study I) determined the glycemic index, while the second session (study II) conducted experiments on exercise performance in the postprandial state. We hypothesized that with the additional amount of PRO, ingestion of CHO + PRO would induce the longest TTE during the exercise performance test. 

## 2. Methods

### 2.1. Ethical Approval and Informed Consent

Institutional ethical approval was obtained before conducting the study (Ethical approval code: 2012B006). Informed consent forms were signed by participants after they completed a pre-exercise medical screening questionnaire. The investigators fully explained the nature of the study, the procedures, and the potential risks. 

### 2.2. Participants

All of the participants were required to have no health complaints and no metabolic disorders, such as Type I and II diabetes, obesity, or Cushing’s syndrome. Those with related complaints or family history of cardiovascular disease (e.g., hypertension, heart attack) were also excluded from the potential participant list, as were smokers. Ten healthy male participants (age 21.70 ± 2.41 years, height 176.60 ± 5.23 cm, body weight 66.58 ± 5.38 kg; and with an endurance running/cycling exercise history of 5.6 ± 1.3 years; V̇O_2max_: 48.1 ± 8.4 mL/kg/min) participated in both study I and study II. 

### 2.3. Prescribed Beverage

In both study I and II, three specific types of sports beverage powders were chosen from the recommendation list for the China national sports teams. They contained oligosaccharides (low molecular weight carbohydrate, L-CHO; CPT^®^, Competitor Co. Ltd., Beijing, China), hydrolyzed starch (high molecular weight carbohydrate, H-CHO; Vitargo^®^, Swecarb AB, Kalmar, Sweden), and whey protein powder with carbohydrate (CHO + PRO, CHO:PRO = 4:1; Endurox^®^ R4, Pacific Health Laboratories, Parsippany, NJ, USA). The powders were dissolved in purified water to a carbohydrate concentration of 8% (Table 1). An artificially sweetened beverage without carbohydrate was chosen as a placebo control (PLA). All of the prescribed solutions were orange flavor with similar textures. In study I, a purified glucose powder was selected as a reference for the glycemic index evaluation. 

### 2.4. Pretest Control

All of the participants were asked to avoid any type of strenuous exercise and to refrain from using caffeine and alcohol during the two days before the experimental day. No food or beverage was allowed after 20:00 p.m. the day before the experiment or on the experimental day. Participants were instructed to consume 500 mL water upon waking in the morning to avoid high viscosity of blood. 

### 2.5. Study I

#### 2.5.1. Experimental Protocol

For the determination of glycemic index, two oral glucose tolerance tests (OGTT) and one glycemic response test were conducted for each sports drink. On the experimental day, participants reported to the lab in the early morning (07:00 a.m.) after undergoing an overnight fast of at least 10 h. During the OGTT, after baseline blood glucose level was measured, approximately 625 mL glucose solution (glucose concentration 8%) containing 50 g purified glucose was ingested within 10 min. Capillary blood glucose was obtained from the fingertip and assayed in 15, 30, 45, 60, 90, and 120 min after the first sip of solution. The same protocol was repeated during the second OGTT. The same procedures were followed for the glycemic response test for sports drinks. Sports drink powders were dissolved to produce CHO concentration of 8%, with the total CHO amount being 50 g. Osmolality of sports drinks was assessed in the Osmometer 2020 (Advanced Instruments, Inc., Norwood, MA, USA), with a standard solution of 290 mOsm/kg, while the pH value was determined with Model 632 (Metrohm, Herisau, Switzerland). Capillary blood glucose concentration was measured in a YSI 1500 (Yellow Spring Ltd., Greene County, OH, USA) instrument with the method of GOD (glucose oxidase).

#### 2.5.2. Calculation of Incremental Area under Curve (IAUC) and GI

The glycemic response curve was determined by placing time points on a horizontal axis and the related glucose level on the vertical axis. The IAUC was calculated based on the glycemic response of different treatments. The specific methods strictly followed the procedures described in previous literature [11]. 

### 2.6. Study II

#### 2.6.1. V̇O_2max_ Test

Prior to the main trial, a preliminary test was conducted to determine the maximum oxygen uptake (V̇O_2max_) of the participants. At the beginning of the test, participants performed a 3-min warm-up on an electrically braked cycle ergometer (Custo Med EC3000, Ottobrunn, Germany) at 50–80 W to prepare for maximal exercise. The initial workload was determined as 100 W and then increased from this initial level by 20 W each minute during the test. Participants were encouraged to maintain their cycling speed at 60 revolutions per minute (rpm). Heart rate was monitored with Polar team package (Polar^®^, Lake Success, NY, USA). Two calibrations for the gas analysis instrument (CORTEX Biophysik GmbH, Leipzig, Germany) were repeated before each gas sample collection. One calibration was a two-point gas calibration for O_2_ and CO_2_ by calibrating both sensors with ambient air. The O_2_ sensor was calibrated with ~15% O_2_, and the CO_2_ sensor with ~5% CO_2_. The other calibration was of the volume transducer with a 3-L calibration syringe. A gas sample was collected and gas analysis was conducted throughout the process of the test. Criteria for determining the peak time point were: (a) respiratory exchange ratio (RER) was greater than 1.10; (b) heart rate (HR) was within 5% of the individual age-predicted maximum heart rate (220-age); (c) the approaching of the oxygen uptake plateau (the last two values agreeing within 2 mL·kg^−1^·min^−1^); (d) the participant was unable to maintain the cadence at 60 rpm for longer than 30 s. VO_2peak_ was determined as the highest VO_2_ reading averaged over two consecutive readings. As the oxygen uptake value linearly increased with the rising of workload, individual exercise intensity during the main trial was determined as the workload in accordance with the 70% of V̇O_2max_ valued. 

#### 2.6.2. Dietary Supplementation

Standard breakfast and lunch were served on the main trial day. Contents for the standardized breakfast were marinated egg in soy sauce (50 g), bread with jam (120 g), and whole fat milk (240 mL); and contents for standardized lunch were steamed rice (320 g), stir-fried onion with pork slices (75 g), stewed beef with potato (120 g), and chicken drumstick (60 g). The standardized foods were weighed with the electrolyte scale. Macro nutrition contents were calculated using “Nutritional Analysis and Management System for Athletes and the Public Catering” software (developed by National Institute of Sports Medicine, Beijing, China). 

#### 2.6.3. Experimental Procedure

No food except water was allowed after 20:00 p.m. the night before the experimental day so as to ensure an overnight fast state. Once participants reported to the lab at 07:00 a.m., a standardized breakfast was provided. One hour following breakfast consumption (08:00 a.m.), an intravenous catheter was inserted (22G, Wellfare, UK) and first blood sample was drawn from the forearm vein for baseline evaluation. After that, pre-exercise body weight was measured. Participants then exercised on the ergometer bicycle for 60 min at 70% V̇O_2max_. One of the prescribed beverages was consumed at 1.8 mL/kg every 15 min during exercise and 10 mL/kg immediately after exercise [7]. Blood samples were obtained from the catheter at 0, 30, and 60 min. A standardized lunch was provided at 12:00 a.m.. Six hours after the first exercise bout (15:00 p.m.), participants conducted the second cycling exercise at 70% V̇O_2max_ until exhaustion. Results for time to exhaustion (TTE) were recorded as seconds (s). During the first 60 min, beverages were provided at the same rate as with the first exercise bout. No drink was served later on, and participants rode at a fix cadence of 60 rpm until unable to maintain this cadence for longer than 30 s. During the TTE test, participants were unaware of the time until they completed the test. Gas samples were collected at 0, 30, and 60 min in both exercise sessions. After 2 min of breathing equilibration, average values for VO_2_ and VCO_2_ were calculated from 3 min of gas collection at each time period. Subjective feelings for perceived exertion were evaluated by the Borg’s rating of perceived exertion scale from 6 (very, very light) to 20 (very, very hard) [12] at 0, 15, 30, 45, and 60 min during both exercise sessions. The CHO and FAT oxidation rate were calculated from the gas analysis result with the previous formula [13]. 


Carbohydrate oxidation = 4.55 VCO_2_ − 3.21 VO_2_ (1)

Fat oxidation = 1.67 VO_2_ − 1.67 VCO_2_ (2)

#### 2.6.4. Blood Measurement Evaluation

Blood glucose and lactate (LA) were evaluated with YSI instruments (YSI 2300 and 1500, Yellow Spring Instrument Co. Ltd., Greene County, OH, USA), respectively. Serum insulin and cortisol were evaluated by the immunofluorescence assay on the Unicel^®^ DxI 800 Access^®^ Immunoassay System and also with evaluation kits provided by the same manufacturer (Beckman Coulter, Brea, CA, USA). The reference range for the insulin kit was 0.21–2100 pmol/L. Intra-assay coefficient of variation (CV) was 2.0%–4.2% and inter-assay CV was 3.1%–5.6%. Assay sensitivity for the cortisol kit was 11.0 nmol/L. Intra-assay CV was 4.4%–6.7%, and an inter-assay CV was 6.4%–7.9%. Serum glucagon was evaluated with an enzyme-linked immunosorbent assay (ELISA) kit (R&D Systems Inc., Minneapolis, MN, USA) according to the instructions provided by the manufacturer. Sensitivity for the glucagon kit was 14.7 pg/mL and reference range was 31.3–2000 pg/mL. Intra-assay CV was 2.7%–3.6%, and inter-assay CV was 5.8%–8.7%. Serum free fatty acid (FFA) was evaluated by the Randox method (Range 0.072–2.24 mmol/L; CV < 5%) on the UniCel^®^ DxC 600 Synchron^®^ Clinical System (Beckman Coulter, Brea, CA, USA). 

### 2.7. Statistical Analysis

All of the continuous data were presented as Mean ± SE and analyzed with SPSS 17.0 for Windows (SPSS Inc. Chicago, IL, USA). For cross-over data with repeat measurements, two-way (treatment × time) repeated measures ANOVA was applied to determine the differences among treatments and time points. For data with unequal variance (IAUC and GI), non-parametric analysis for multiple samples (Kruskal-Wallis test) was applied. Significance level was set at 0.05.

## 3. Results

### 3.1. Study I

#### 3.1.1. Blood Glucose

**Figure 1:**
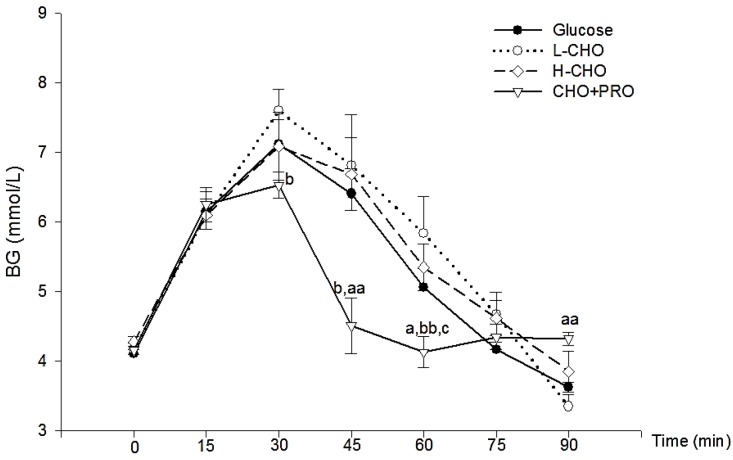
Blood glucose (BG) change after ingestion of different solutions. a: vs. Glucose, *p* < 0.05; aa: vs. Glucose, *p* < 0.01; b: vs. L-CHO, *p* < 0.05; bb: vs. L-CHO, *p* < 0.01; c: vs. H-CHO, *p* < 0.05. CHO: carbohydrate; H-CHO high molecular weight CHO; L-CHO low molecular weight CHO; PRO: protein.

Blood glucose (BG) changes after ingestion of the sports drink and glucose solutions are shown in Figure 1. No differences were observed between the solutions at baseline. However, BG of CHO + PRO at 30 min was significantly lower, compared with L-CHO (*p* < 0.05). In addition, at 45 min and 60 min, BG of L-CHO and reference glucose were higher than CHO + PRO, while at 90 min, BG of CHO + PRO was significantly higher than reference glucose (*p* < 0.01) (Figure 1). 

#### 3.1.2. IAUC and GI

The IAUC and GI of the reference glucose solution and the three sports drinks are shown in Table 2. With standardized glucose solution as the reference, both L-CHO and H-CHO were categorized as high GI solution, while CHO + PRO was classified as medium GI. 

### 3.2.Study II

#### 3.2.1. Dietary Supplementation

Participants were provided with a standardized breakfast with total energy content of 556.5 kcal, with 55.65% from CHO, 16.52% from PRO, and 27.83% from fats (lipids). For the standardized lunch provided between the exercise bouts, the total energy content was 852.3 kcal, with 44.33% from CHO, 17.90% from PRO, and 37.77% from FAT (Table 3). 

#### 3.2.2. Blood Measurements

**Figure 2:**
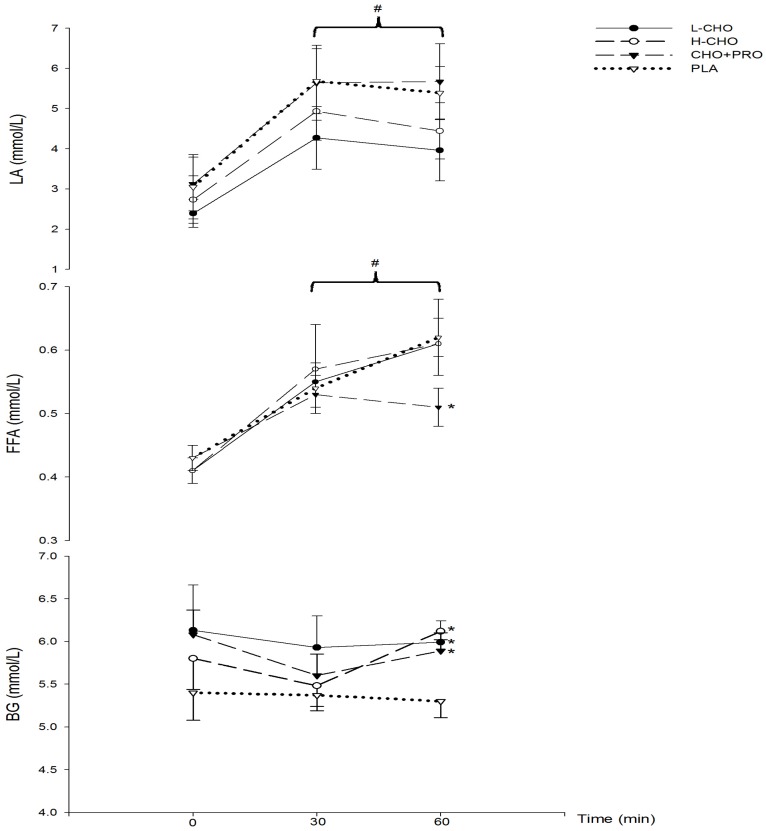
Changes of blood glucose (BG), lactate (LA) and free fatty acid (FFA) during the first exercise session (^#^: vs. 0 min, *p* < 0.05; *: vs. placebo (PLA), *p* < 0.05).

In treatments with CHO, blood glucose (BG) was significantly higher than PLA at the end of exercise (60 min) (*p* < 0.05). Compared with baseline (0 min), free fatty acids significantly increased in all treatment groups at 30 min and 60 min (*p* < 0.05). In treatments with CHO, compared to PLA, FFA was significantly lower at 60 min (*p* < 0.05). At 30 and 60 min, lactate increased significantly in all groups (*p* < 0.05); however, no significant difference was found between the treatments (*p* > 0.05) (Figure 2). At 90 min, insulin in PLA was significantly lower than in other treatments (*p* < 0.05). Apart from the CHO + PRO group, insulin levels at 30 and 60 min were significantly lower than baseline (*p* < 0.05). No significant differences were found in glucagon and cortisol among the treatments (*p* > 0.05) (Table 4). 

#### 3.2.3. Substrate Utilization, Subjective Feelings, and Subsequent Performance

**Figure 3:**
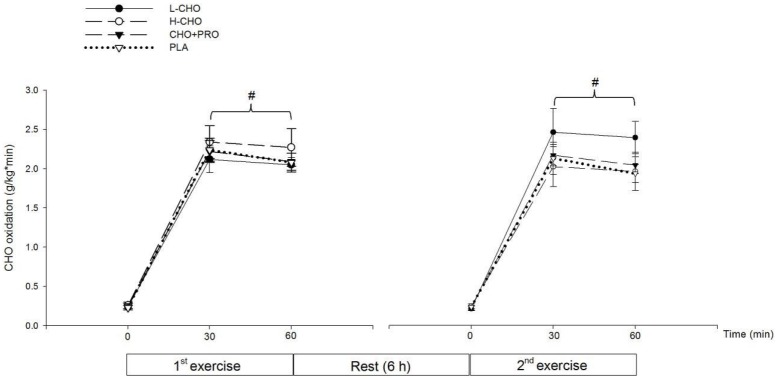
CHO oxidation rate in the first and second exercise sessions in study II (^#^: vs. 0 min in the same exercise bout, *p* < 0.05).

**Figure 4:**
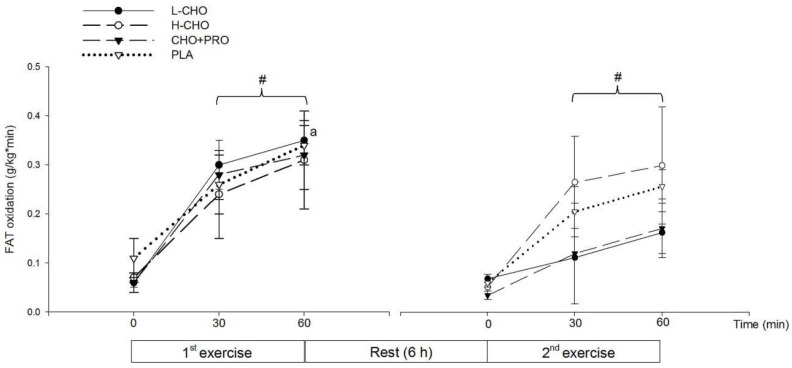
FAT oxidation rate in the first and second exercise sessions in study II (^#^: vs. 0 min in the same exercise bout, *p* < 0.05; a: vs. 2nd exercise session in 60 min, *p* < 0.05).

**Figure 5:**
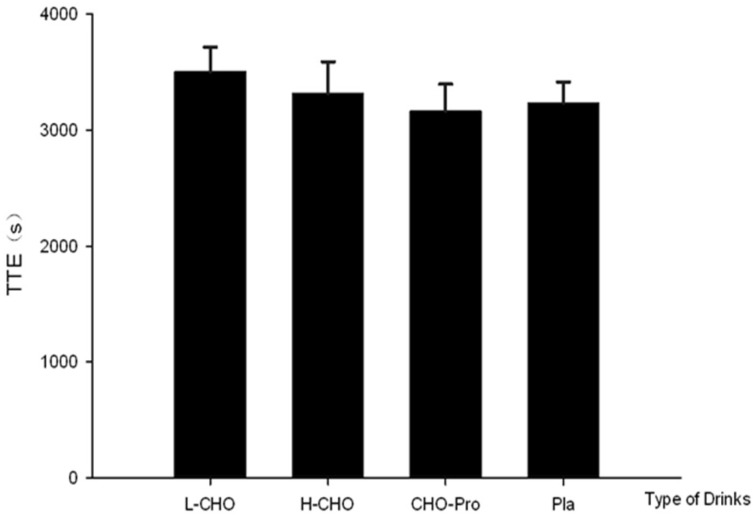
Time to exhaustion (TTE) following treatments.

CHO oxidation rate significantly increased along the exercise, following the ingestion of all treatments (*p* < 0.05) (Figure 3). FAT oxidation rate in the first exercise session was significantly higher than in the second session at 60 min, following L-CHO treatment (*p* < 0.05) (Figure 4). No significant difference was found among treatments regarding RPE at each time point (*p* > 0.05) (Table 5). Results of time to exhaustion (TTE, s) were not significantly different among treatments (L-CHO vs. H-CHO vs. CHO + PRO vs. PLA: 3505.40 ± 210.53 vs. 3317.67 ± 273.22 vs. 3164.33 ± 232.99 vs. 3237.33 ± 179.77 s, *p* > 0.05) (Figure 5). 

## 4. Discussion

In the present study, we investigated the effects of commercially available sports beverages with various compositions (L-CHO, H-CHO, CHO + PRO) on substrate metabolism and subsequent performance in postprandial endurance exercise. 

In study I, both L-CHO and H-CHO beverages were found to possess a high glycemic index (L-CHO GI: 117.70; H-CHO GI: 105.50), while the GI level in the CHO + PRO beverage was medium (GI: 67.23). Previous studies have indicated that additional PRO ingestion induces higher insulin production than CHO alone [14,15]. With the increase in insulin, the glucose concentration in peripheral venous blood (i.e., blood glucose—BG) decreased, and therefore induced a lower glycemic response following CHO + PRO. Moreover, insulin increases glucose transport through the cell membrane. This allows more glucose to enter into cells for storage or utilization [16]. All of these findings suggest that ingestion of CHO + PRO may provide the greatest amount of energy substrate during exercise. 

During exercise (study II), BG following L-CHO, H-CHO, and CHO + PRO was significantly higher compared to PLA, while BG at 60 min following PLA remained unchanged compared to baseline (Table 4). As a result, insulin concentration was the lowest at 60 min following PLA. Moreover, it also remained unchanged following CHO + PRO ingestion, while it decreased significantly following L-CHO, H-CHO, and PLA. These results were also consistent with previous resting studies [14,15]. However, no significant difference was found among treatments and time points in glucagon concentration (Table 4). In studies with an overnight fast state, nutrition supplementation with high GI induced more intensive glycemic and insulin response than supplementation with lower GI. High GI foods can be absorbed more rapidly, and therefore provide quick energy for exercise [16]. Results of the present study indicate that the beverage GI did not influence the blood glucose availability in post-prandial state. CHO utilization during exercise was not influenced by either energy supplement or the GI of the sports beverages. In terms of FAT utilization, FFA increased significantly during exercise in all treatments groups in spite of adequate CHO supply. With the greatest amount of exogenous energy supplementation, FFA following CHO + PRO was significantly lower than PLA. No significant difference was found among L-CHO, H-CHO, and PLA. This result is in accordance with the change of insulin following treatments. As insulin concentration remained following CHO + PRO, the lipolysis process was also suppressed to the greatest extent among all of the treatments. As a result, there was a lower FFA level following CHO + PRO than in the other groups. Meanwhile, increasing insulin is associated with decreasing endogenous glucose production [17]. This indicates that supplementation of CHO + PRO would be potentially beneficial during periods of exercise longer than the 60 min examined in the present study. However, whether there is any effect on the blood glucose, insulin, and glucagon concentration following treatment during exercise longer than 60 min remains to be investigated. 

A subsequent TTE exercise performance test was conducted six hours after the first exercise session. During the performance test in the present study, the ingestion protocol—namely L-CHO, H-CHO, CHO + PRO and PLA—after standardized meal did not influence the exercise outcome. No significant difference was found among CHO and FAT oxidation rates following different treatments (Figure 5). This result contradicts our initial hypothesis that CHO + PRO would induce the best outcome in TTE test due to the greatest energy supplementation as well as the potential physiological benefits following PRO ingestion. However, this result is partly consistent with one previous study. It was shown CHO consumption in the postprandial state did not influence the performance outcome in 16.1 km cycling time trial in either temperate (18 °C) or hot (32 °C) environment [18]. Exercise performance is influenced by blood glucose and CHO oxidation rate [19,20]. In addition, enhancement of CHO availability was found to be associated with the decreasing rating of perceived exertion [21]. Similar exercise performance and perceived exertion (Table 5) outcome in the present study were therefore attributed to similar CHO availability, indicated by blood glucose and CHO oxidation rate, following those treatments. 

In addition, FAT oxidation rate in the second exercise session was lower than that in the first session following the ingestion of L-CHO. Although not significant, FAT oxidation rates in the 60 min of the second exercise session tended to be lower than the first following CHO + PRO ingestion, while the FAT utilization was maintained following the ingestion of H-CHO and PLA. On the one hand, lower FAT oxidation rates indicated a suppressing effect of the FAT utilization during the second exercise session. This could be attributed to the greatest GI of the L-CHO beverage, as it could be absorbed most rapidly among the treatments. It can be speculated that both the CHO + PRO and L-CHO could induce a better outcome in TTE than H-CHO and PLA if the participants were able to exercise for a longer time, and CHO + PRO could induce the best outcome among treatments when the duration was extended. This makes it of value to conduct future study in athletes with higher fitness level, such as elite endurance athletes at national levels. In the present study, the performance result from the present study also indicated that neither the substrate utilization nor the exercise performance was influenced by the energy density or the GI of treatments in the postprandial state.

In summary, the CHO sports beverage with additional PRO maintains insulin production during endurance cycling at 70% V̇O_2max_ in the postprandial state. L-CHO sports beverage suppresses FAT utilization during the subsequent exercise performance test. Subsequent exercise performance (evaluated by TTE) in the present study was not influenced by the type of CHO or the addition of PRO in the selected commercially available sports beverages. 

## Figures and Tables

**Table 1:** Ingredients of prescribed sports beverages. CHO: carbohydrate; PRO: protein.

Ingredients information	L-CHO	H-CHO	CHO + PRO
Energy density (kJ/100 g)	1500	1550	1581
CHO (g/100 g)	90	92	70.3
FAT (g/100 g)	<0.5	<0.5	<0.5
PRO (g/100 g)	0	0	27
CHO molecular weight (g/mol)	<1500	500,000~750,000	337.8

**Table 2:** Incremental area under the curve (IAUC) and glycemic index (GI) of prescribed sports beverage.

Type of Solutions	IAUC	GI
Glucose	143.16 ± 13.23	100
L-CHO	178.97 ± 36.94	117.70 ± 14.25
H-CHO	149.50 ± 19.20	105.50 ± 12.82
CHO + PRO	94.74 ± 10.30	67.23 ± 5.88

**Table 3:** Composition of macronutrients in standardized meals.

Standardized Meals	Characteristics of Macronutrients	CHO	PRO	FAT
Breakfast	Content (g)	77.4	23.0	8.3
	Energy (%)	55.65	16.52	27.83
	Total energy (kcal)	556.5		
Lunch	Content (g)	95.1	38.2	35.8
	Energy (%)	44.33	17.90	37.77
	Total energy (kcal)	852.3		

**Table 4:** Blood hormonal change during exercise following treatments.

Blood Hormonal Measurements	0 min	30 min	60 min
*Insulin (pmol/L)*			
L-CHO	224.13 ± 30.84	67.10 ± 27.31 ^#^	104.36 ± 22.49 *^,#^
H-CHO	245.45 ± 43.15	81.97 ± 11.91 ^#^	83.26 ± 13.15 *^,#^
CHO + PRO	169.86 ± 31.56	103.64 ± 21.47	107.09 ± 21.46 *
PLA	188.74 ± 31.78	61.27 ± 11.22 ^#^	27.38 ± 3.50 ^#^
*Glucagon (pg/mL)*			
L-CHO	41.85 ± 12.66	51.00 ± 9.45	56.97 ± 8.43
H-CHO	54.91 ± 8.50	47.30 ± 13.87	51.58 ± 13.08
CHO + PRO	33.16 ± 9.29	52.60 ± 15.70	62.72 ± 22.11
PLA	53.24 ± 10.56	67.40 ± 12.31	74.89 ± 11.13
*Cortisol (nmol/L)*			
L-CHO	15.36 ± 1.60	17.32 ± 3.31	16.09 ± 1.27
H-CHO	14.68 ± 1.90	15.79 ± 1.74	14.63 ± 1.09
CHO + PRO	20.99 ± 7.79	15.03 ± 1.09	16.07 ± 1.47
PLA	13.59 ± 1.16	13.70 ± 1.11	14.49 ± 1.27

^#^: vs. 0 min, *p* < 0.05; *: vs. PLA, *p* < 0.05.

**Table 5:** Rating of perceived exertion following treatments.

Treatments	Sample size (*N*)	0 min	15 min	30 min	45 min	60 min
	*1st exercise*					
	*N*	10	10	10	10	10
L-CHO		8.10 ± 0.52	12.80 ± 0.49 ^#^	14.80 ± 0.94 ^#^	16.40 ± 0.76 ^#^	17.30 ± 0.86 ^#^
H-CHO		7.90 ± 0.48	12.60 ± 0.52 ^#^	14.00 ± 0.59 ^#^	14.20 ± 0.61 ^#^	13.90 ± 1.03 ^#^
CHO-PRO		8.70 ± 0.66	12.50 ± 0.68 ^#^	14.20 ± 0.78 ^#^	15.30 ± 0.86 ^#^	15.70 ± 0.96 ^#^
PLA		9.00 ± 0.59	12.60 ± 0.47 ^#^	13.80 ± 0.29 ^#^	15.00 ± 0.51 ^#^	15.20 ± 0.61 ^#^
	*2nd exercise*					
L-CHO	*N*	10	10	10	8	7
		8.78 ± 0.62	12.89 ± 0.75 ^#^	14.56 ± 0.91 ^#^	16.12 ± 0.79 ^#^	17.42 ± 0.97 ^#^
H-CHO	*N*	10	10	10	7	5
		8.56 ± 0.82	12.22 ± 0.55 ^#^	15.11 ± 0.48 ^#^	16.29 ± 0.94 ^#^	17.40 ± 1.29 ^#^
CHO-PRO	*N*	10	10	10	9	7
		8.90 ± 0.60	14.10 ± 0.48 ^#^	15.60 ± 0.73 ^#^	16.55 ± 0.73 ^#^	17.14 ± 0.83 ^#^
PLA	*N*	10	10	10	9	8
		9.00 ± 0.59	12.80 ± 0.61 ^#^	14.40 ± 0.65 ^#,^*	14.67 ± 0.73 ^#^	15.75 ± 0.76 ^#^

1st exercise = the first exercise session; 2nd exercise = the second exercise session; ^#^: vs. 0 min, *p* < 0.05.; *: v.s. 15 min, *p* < 0.05.
